# When salt is needed to grow: Questions

**DOI:** 10.1007/s00467-020-04639-8

**Published:** 2020-08-10

**Authors:** Ester Conversano, Sara Romano, Andrea Taddio, Flavio Faletra, Davide Zanon, Egidio Barbi, Marco Pennesi

**Affiliations:** 1 Department of Medicine, Surgery, and Health Sciences, University of Trieste, Trieste, Italy; 2 Paediatric Department, Institute for Maternal and Child Health - IRCCS “Burlo Garofolo”, Trieste, Italy; 3 Genetic Department, Institute for Maternal and Child Health - IRCCS “Burlo Garofolo”, Trieste, Italy; 4 Pharmacy and Clinical Pharmacology Department, Institute for Maternal and Child Health - IRCCS “Burlo Garofolo”, Trieste, Italy

**Keywords:** Child, Metabolic acidosis, Hyperkalaemia, Hyponatraemia, Failure to thrive

## Case summary

A 50-day-old girl was referred for a history of recurrent vomiting, poor feeding and moderate failure to thrive. She was born by a caesarean section at 41 + 5 weeks of gestation, from non-consanguineous parents. Her birth weight was 3430 g and she was fed with breast milk and formula. Upon admission, her weight was 3700 g (3° percentile) and blood pressure was normal. Physical examination showed mild dystrophy with poor representation of subcutaneous fat, normal external genitalia and age-appropriate psychomotor development.

Laboratory tests showed mild hyponatraemia (130 mEq/L) and hyperkalaemia (6 mEq/L), normal plasma creatinine (0.26 mg/dl) and metabolic acidosis (pH 7.23, pCO2 53 mmHg, HCO3^-^ 21.8 mmol/L, lactic acid 7.5 mmol/L). Potassium and sodium urinary fractional excretion were 23% and 0.98%, respectively. In the urine sample, no proteinuria, white blood cells, bacteria or haematuria were detected. Abdominal ultrasound results were normal.

Due to the acidosis with persistence of poor feeding and vomiting, a hydration with an intravenous physiological solution was empirically started. While metabolic acidosis was resolved with the infusion, hyponatraemia and hyperkalaemia persisted. Further analysis revealed a significant increase of plasma renin activity and aldosterone (respectively, > 500 μUI/mL, normal value 4 44; > 1000 ng/dL, normal value 30–50).

## Questions

1. What is the most likely diagnosis?
2. What would be an alternative diagnosis?
3. What is the treatment and prognosis of the disease?

